# Anti-fouling Coatings of Poly(dimethylsiloxane) Devices for Biological and Biomedical Applications

**DOI:** 10.1007/s40846-015-0029-4

**Published:** 2015-04-01

**Authors:** Hongbin Zhang, Mu Chiao

**Affiliations:** Department of Mechanical Engineering, University of British Columbia, Vancouver, Canada

**Keywords:** Anti-fouling, Surface modification, Poly(dimethylsiloxane) (PDMS), Biomedical devices

## Abstract

Fouling initiated by nonspecific protein adsorption is a great challenge in biomedical applications, including biosensors, bioanalytical devices, and implants. Poly(dimethylsiloxane) (PDMS), a popular material with many attractive properties for device fabrication in the biomedical field, suffers serious fouling problems from protein adsorption due to its hydrophobic nature, which limits the practical use of PDMS-based devices. Effort has been made to develop biocompatible materials for anti-fouling coatings of PDMS. In this review, typical nonfouling materials for PDMS coatings are introduced and the associated basic anti-fouling mechanisms, including the steric repulsion mechanism and the hydration layer mechanism, are described. Understanding the relationships between the characteristics of coating materials and the accompanying anti-fouling mechanisms is critical for preparing PDMS coatings with desirable anti-fouling properties.

## Introduction

Over the past few decades, poly(dimethylsiloxane) (PDMS) has been widely used in biomedical applications such as medical/surgical implants, pacemaker encapsulants, catheters and contact lenses [1–3]. More recently, the use of PDMS has been extended to analytical chemistry, drug delivery, biological synthesis and analysis, disease diagnostics, DNA sequencing, and biosensors [4–8]. The popularity of PDMS is driven by its attractive features such as biocompatibility, low toxicity, optical transparency, elastomeric properties, gas permeability, ease of fabrication, and low manufacturing costs [9]. However, owing to the intrinsic hydrophobicity of PDMS, PDMS-based devices suffer low wettability and biofouling problems from nonspecific protein/hydrophobic analyte adsorption and cell/bacterial adhesion. As a result, safety issues arise when PDMS is used in a blood contact environment and low performance may be a concern for long-term application in microfluidics [10]. To overcome the above-mentioned limitations, many studies, involving both physical and chemical methods, have been conducted to render the PDMS surface hydrophilic and give it anti-fouling capability. Physical methods change the state of the PDMS surface through physical interactional processes, such as the adsorption of coating materials onto the PDMS surface via hydrophobic or electrostatic interactions, and surface activation by physical treatment of oxygen plasma, ozone, or ultraviolet (UV) light. However, the anti-fouling property of PDMS via physical modification is temporary, with the surface usually undergoing hydrophobic recovery after a period of time. With chemical modification, covalent bonds are formed between a PDMS substrate and coating materials, making the surface relatively stable and giving it a permanent anti-fouling property. In general, chemical modification is a complex process with several reagents and multiple synthetic and purge steps, which may pose challenges for large-scale production in some cases.

Several excellent reviews [10–14] have covered existing surface modification strategies for fabricating nonfouling PDMS surfaces through both physical and chemical methods. The present review focuses on the structures and mechanisms of anti-fouling coating polymers. It is known that polymer chain length plays an important role in anti-biofouling mechanisms. For short-chain-length polymers, a hydration layer forms due to their hydrophilicity, providing protein resistance. For long-chain-length materials, in addition to a hydration layer, steric repulsion from the flexible polymer chains is believed to contribute to anti-fouling properties. In order to tune the hydration layer and the steric repulsion, several types of polymers have been developed, including poly(ethylene glycol) (PEG)-based materials and polyzwitterionic materials. This paper reviews recent progress on anti-fouling coatings for PDMS surfaces.

## Protein Fouling and Anti-fouling Principles

Biofouling is the accumulation of an unwanted substance (proteins and other analytes) or organisms (cells and bacteria) on the wetted surface of host materials [15]. The adsorption of contaminating matter can alter the performance of biomedical implants and devices and lead to patient infection, shortened durability, and increased healthcare cost from replacement of devices [15]. Among various types of fouling, protein fouling (nonspecific protein adsorption) is a dominant factor in the failure of many biomedical implants and devices [16]. Nonspecific protein adsorption at the tissue-device interface is considered to be the initial event when a device is exposed to biological media. The adsorbed proteins can reduce the sensitivity of in vitro diagnostics such as immunological assays, lead to several deleterious host responses (inflammation and thrombosis), and modify therapeutic properties like the drug release profile. It has been reported [17] that fibrinogen adsorption, even at 10 ng·cm^−2^, can initiate full-scale blood platelet adhesion, leading to medical device failure and adverse patient outcomes. Furthermore, the formation of a protein layer on medical devices facilitates bacterial attachment and subsequent biofilm formation, which dramatically increases the risk of infectious diseases. For example, in ophthalmic applications, such as contact lens, tear protein adsorption on the lens can cause patient discomfort and increases chances of infection [18–20].

**Fig. 1:**
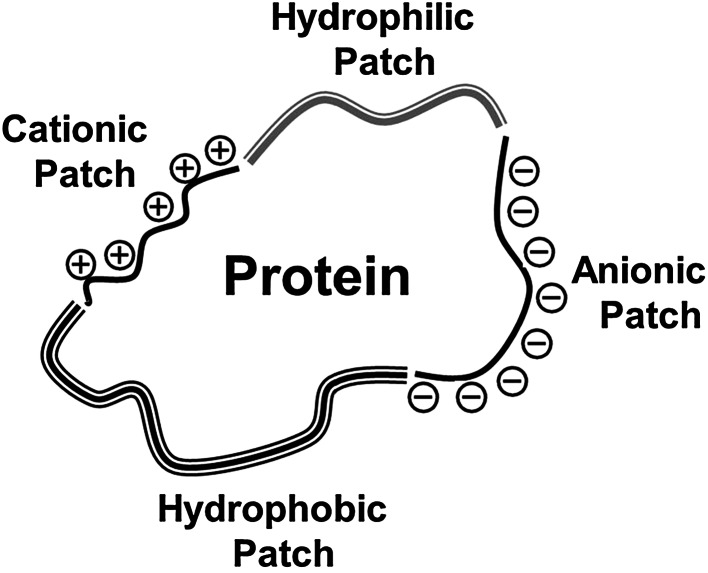
Schematic diagram illustrating the non-homogeneous nature of a protein surface

In general, the basic goal of anti-fouling coatings is to minimize the intermolecular forces and interactions between contaminating matter and the surface of the host substrate so that adhered biomacromolecules and cells can be easily detached and released under low shear stresses. However, the surface fouling process is complicated, especially for biomacromolecules with complex compositions and molecular structures such as proteins. Most proteins comprise some polypeptide chains with different sequences of amino acid residues or primary structures. These residual amino acids are prone to link together through hydrogen bonding, forming secondary structures of proteins, such as α-helix and β-sheet structures and turns. In addition, interactions of polypeptide chains, such as salt bridges, hydrogen bonds, disulfide bonds, and electrostatic interactions, further cause proteins to fold into unique three-dimensional (3-D) structures (tertiary structures). Finally, these 3-D polypeptides or proteins can further associate and generate quaternary structures. As shown in Fig. 1, a protein surface may simultaneously contain hydrophobic, hydrophilic, cationic, and anionic regions. Moreover, the distribution and proportion of these different regions on the protein surface changes with environmental factors such as pH, temperature, an ionic strength. The adsorption of proteins may thus result from hydrogen bonding, electrostatic, charge-transfer, and/or hydrophobic interactions, depending on the surface properties of the host [21].

For pure-PDMS devices, hydrophobic interactions are the dominating driving force of surface protein adsorption, especially in aqueous environments. Hydrophobic interactions between PDMS substrates and proteins can further cause conformational changes and denaturation of protein molecules, resulting in irreversible adsorption. Therefore, enhancing the hydrophilicity of PDMS with proper materials is a direct and effective way to minimize undesirable interaction with proteins, and thus to decrease surface fouling. Since protein fouling is such a common and intractable problem for most PDMS-based biomedical implants and devices, this review mainly focuses on protein-fouling-resistant coatings.

## Anti-fouling Coatings for PDMS Devices

On the basis of empirically derived design criteria, polymers with anti-fouling properties should be hydrophilic and electrically neutral, and should have hydrogen bond acceptors but no hydrogen bond donors [22]. However, long-term anti-fouling ability of materials is still difficult to achieve. Significant effort based on theory and trial-and-error approaches has been invested in the search for optimal anti-fouling materials, based on which several classes of materials have been developed and proved to have good protein resistance properties.

### PEO/PEG-Based Coatings

**Fig. 2:**
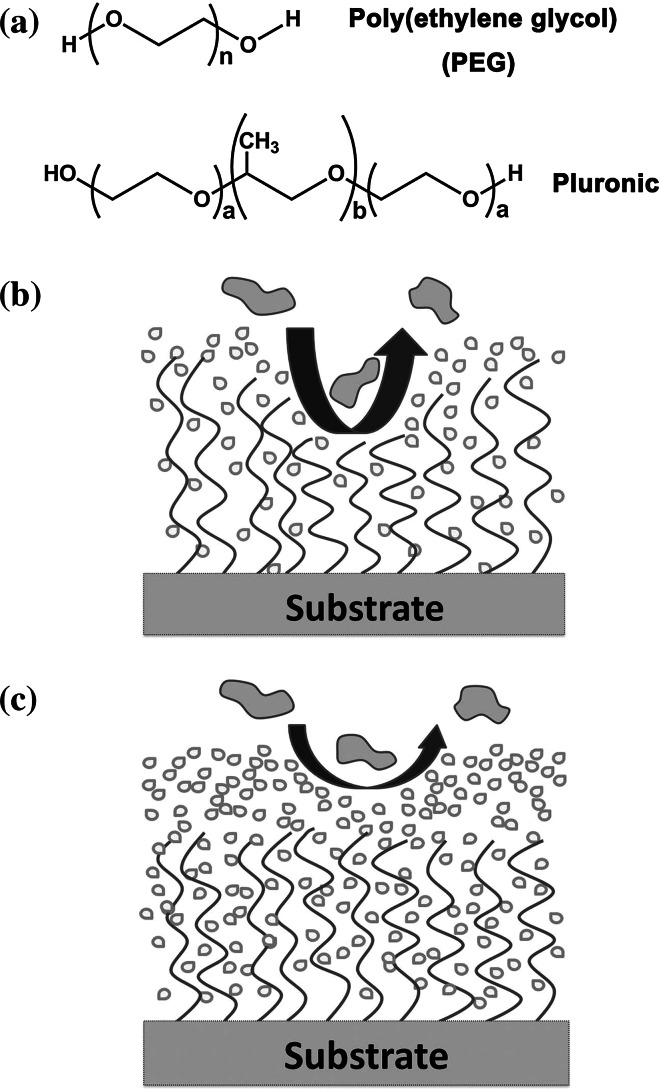
**a** Chemical structures of poly(ethylene glycol) (PEG) and a Pluronic polymer. Illustrations of **b** steric repulsion mechanism of polymers with flexible long chains and **c** hydration layer mechanism of hydrophilic polymers for surface resistance to nonspecific protein adsorption

Poly(ethylene oxide) (PEO) and PEG are basically the same polymer, having the same repeating unit (–CH_2_CH_2_–O–) (Fig. 2a), but derived from different monomers and polymerization methods. PEG has a simple structure and exhibits many desirable properties, such as hydrophilicity and nontoxicity [23–25]. It has been widely used in surface modification [26, 27]. Furthermore, PEG is a well known anti-fouling material with few possibilities to bind with proteins due to its weakly basic ether linkages and its low value of polymer-water interfacial energy (below 5 mJ·m^−2^) [28].

PEG can be attached to PDMS by physical/chemical adsorption methods, direct covalent attachment, and graft copolymerization. Some PEG-based coatings are summarized in Table 1. In physical adsorption methods, PEG usually copolymerizes with other macromolecules that interact with the PDMS surface through electrostatic or hydrophobic interactions. In Lee an Vörös’ work [29], a polycation-PEG graft copolymer, poly(l-lysine)-graft-PEG (PLL-g-PEG) could be easily adsorbed on the PDMS surface treated by oxygen plasma. The polycationic PLL backbone was attracted onto the negatively charged oxidized PDMS surface with an extended structure of PEG chains to the aqueous environment, forming an excellent protein-resistant solid/liquid interface. By using electrostatic layer-by-layer assembly, Makamba et al. introduced end-functionalized PEG into a polyelectrolyte multilayer on a passive PDMS channel [30]. The functionalized PEG was then crosslinked with polyelectrolytes via carbodiimide coupling between amine and carboxyl groups to produce stable, hydrophilic, protein-resistant coatings that resist hydrophobicity recovery in air. Amphiphilic PEG-copolymers containing molecular blocks of various compositions have been prepared for modifying the surface properties of biomaterials in various applications [31]. Pluronic is a class of powerful and popular surfactants for dynamic coating in capillary electrophoresis. Pluronic surfactants have a triblock copolymer structure of PEO-poly(propylene oxide)-PEO (PEO-PPO-PEO, Fig. 2a). This type of polymer can directly attach to various hydrophobic materials through spontaneous surface adsorption of the hydrophobic PPO moieties [32]. Pluronic surfactants have been used as coating materials for PDMS microchannels to reduce electroosmotic flow [33], and to maintain a steady protein level with low serum protein adsorption for a PDMS microbioreactor in a culture system [34]. Apart from Pluronic adsorption, gradient-induced migration of embedded Pluronic molecules in PDMS was studied as a surface modification method for PDMS [35]. Since the solubility of Pluronic in water is much higher than that in the PDMS matrix, when PDMS contacts water, the hydrophilic PEO moieties of embedded Pluronic tend to migrate to the interface of water and PDMS while hydrophobic PPO tend to stay on the surface or be partly embedded in the PDMS. Thus, after a certain time of Pluronic migration, a hydrophilic and protein-resistant PDMS surface is obtained.

**Table 1:** PEG/PEO-based anti-fouling coatings for PDMS devices

Coating methods	Chemical structures	Description	Ref.
Physical adsorption		Materials were synthesized and adsorbed on PDMS by electrostatic interaction, showing stable protein resistance to human fibrinogen for 12 weeks	[29]
		Commercially available materials were attached to PDMS by hydrophobic interaction	[33] [34]
Chemical adsorption/covalent bonding		Materials were synthesized and self-assembled on substrates through silane coupling. Resistance to nonspecific protein adsorption and cell adhesion was observed	[38]
		Commercially available materials were allowed to interact with PDMS to form covalent attachment to reduce protein adsorption	[39–42]
		Commercially available materials were used as a coating formed by platinum-catalyzed hydrosilylation of PEG with Si–H groups on PDMS	[43] [44]
		Commercially available PEG was immobilized on PDMS through an amine-NHS reaction which is facile and environmentally friendly	[45] [46]
		Materials were easily prepared and reacted with epoxy groups on PDMS surface	[47]
		Commercially available materials were grafted onto PDMS through SI-ATRP without UV/ozone pretreatment steps	[48]

Coating the PDMS surface with physisorption strategies is simple and quick in experiments. However, these coating layers are easily destroyed or removed under influence from the environment by factors such as temperature, mechanical factors, and solvolytic influence due to their weak adsorption affinity to the substrate surface. A lot of research effort has been devoted to developing methods to improve the durability and resistance to biological contamination of PEG coatings. The use of self-assembled monolayers (SAMs) is one such effective technique. SAMs form from organic molecules spontaneously anchoring onto a reactive solid surface [36, 37]. Materials for SAMs commonly consist of a head group which can be adsorbed on and covalently connect to the substrate, and a tail with a special structure or functional groups to confer desired properties to the modified surface. In general, alkoxysilanes, such as trichlorosilane, triethoxysilane and trimethoxysilane derivatives with functional molecular structures at their ends, are commonly employed for SAM coatings on PDMS surfaces through silanization [37]. In the work of Jon et al. [38], a random copolymer composed of trialkoxysilane as an anchor part and PEG as a function part, was synthesized and coated onto an oxygen-plasma-treated Si/SiO_2_ surface. The SAM coating led to significant reductions (up to 98 %) in protein adsorption of insulin, lysozyme, and fibrinogen compared to that for uncoated Si/SiO_2_ wafers. In addition, cell adhesion of 3T3 fibroblasts was suppressed on modified glass substrates. Papra et al. modified the microchannels of PDMS and glass using commercially available PEG-silanes to increase the protein resistance for assisting protein patterning in microfluidic networks [39, 40]. Using similar adsorbate materials, Chen et al. prepared protein-resistant PDMS elastomers by incorporation of mono- or bis(triethoxysilyl)PEO with different molecular weights (MWs) in the rubber during rubber formation [41, 42]. By doing so, the PEO chains were covalently bound to the backbone of PDMS and could migrate to the surface when exposed to an aqueous environment. Mono-functional PEO with the lowest MW was found to be the most protein-repellent. This was probably because the free PEO ends more easily migrate to the aqueous interface and consequently form a higher surface density of PEO chains on the PDMS surface compared with that of bifunctional PEO with a higher MW [41, 42].

In addition to silanization, direct covalent grafting of PEG/PEO to the PDMS surface via other chemical linker groups can create a protein-resistant surface. These covalent linking reactions between PDMS and PEO include platinum-catalyzed hydrosilylation of PDMS-Si-H and PEO–CH=CH_2_ [43, 44], and crosslinking between primary amines (–NH2) and N-hydroxysuccinimide (NHS) esters [45, 46] and epoxides [47]. The amine-related crosslinking reactions are also very commonly used for labeling peptides and proteins in biology research. In principle, a pre-functionalized PDMS surface with reactive groups that can crosslink with PEG-containing polymers could be used to design covalently attached anti-fouling coatings for PDMS devices. Tugulu and Klok successfully grew nonfouling PEG brushes on a PDMS surface by surface-initiated atom transfer radical polymerization (SI-ATRP), in which no UV/ozone pretreatment steps were involved [48].

Decreasing surface tension or energy by incorporating fluorinated PEG into the PDMS matrix has been used to create low-fouling surfaces for marine anti-fouling coatings to prevent contamination of microorganisms [49, 50]. Because of the low surface tension, the adhesion of foulants is weak. The foulants can thus be released under the hydrodynamic forces created when a vessel moves or by cleaning procedures.

In parallel to extensive experimental efforts, studies seeking to theoretically explain the high protein resistance of PEG have also been reported [51–61]. In early studies of Jeon et al. [51, 52], the flexibility of the PEG chain that extends from a hydrophobic substrate to a water environment was suggested to play an important role in protein resistance. They suggested that when proteins move towards the substrate surface, the compression of PEG chains can result in a repulsive elastic force to resist protein adsorption (Fig. 2b). The magnitude of the resulting steric repulsion depends on the surface density and length of PEG chains. Increasing the surface density and chain length of the polymer would lead to greater protein resistance. As most hydrophilic polymeric materials have some protein resistance, the hydration layer near the coating surface is thought to be the primary cause for the anti-fouling property of water-soluble polymers [59–61]. A tightly bound water layer around a coating material can work as a physical and energetic barrier against the adsorption of proteins on the surface (Fig. 2c). In the case of PEG, when its chains stretch in water, the abundant water molecules residing on and/or penetrating into the coatings form surface-bound water molecules via hydrogen bonds. When proteins approach a PEG-modified surface, the expulsion of water molecules from the hydrated polymer chains creates a thermodynamically unfavorable dehydration entropic effect [62]. This could be the reason for surfaces coated with short-chain oligo(ethylene glycol) (OEG) polymers showing remarkable protein resistance [63–65]. Therefore, anti-fouling performance depends on the hydration capability of coating materials and the hydration extent on the surface, both of which are mainly determined by the physicochemical properties of the coating materials and factors in the environment [66]. Since a hydrogen bond is not stable, PEG coatings could be changed from nonfouling to fouling by reducing their surface hydration by increasing hydrophobicity when copolymerized with hydrophobic monomers [60] or increasing environmental temperature [67].

In summary, it is believed that both steric repulsion and a hydration layer are the primary mechanisms associated with protein resistance of PEG chains [68, 69], and only when these two mechanisms work together can the optimal anti-fouling capability be achieved [70]. However, available theoretical molecular simulations and assumptions are impossible to cover all the factors that influence surface fouling. For instance, in previous theoretical works, proteins were simply treated as finite spherical particles and polymers were treated as random coils. However, as mentioned previously, different proteins have different compositions, MWs and conformations. The molecular conformation of a given protein can even vary in different environments. Therefore, although various strategies have been developed for tethering PEG on different types of substrate in recent years, more research is still needed to understand the protein resistance of PEG and other anti-fouling materials.

### Polyzwitterion-Based Coatings

Zwitterions are a class of material that has an equal amount of positively and negatively charged groups within a single molecule, and thus exhibits overall electrical neutrality. In 1977, Zwaal et al. [71] found that negatively charged phospholipids, which compose the inner membrane of erythrocyte cells, are thrombogenic. In contrast, zwitterionic lipid phosphatidylcholines which are the major component of the outer membrane, are non-thrombogenic. Since then, approaches based on biomimicry have been used to develop new biomaterials with structures similar to those of lipid components in cell biomembranes. Phosphorylcholine (PC)-containing polymers were first synthesized by Chapman et al. [72]. Surface coated with such materials have been prepared and demonstrated to be non-thrombogenic [73, 74]. Further development that involves a PC headgroup or variants, such as sulfobetaine (SB) and carboxybetaine (CB), promises better anti-fouling and biocompatibility.

**Fig. 3:**
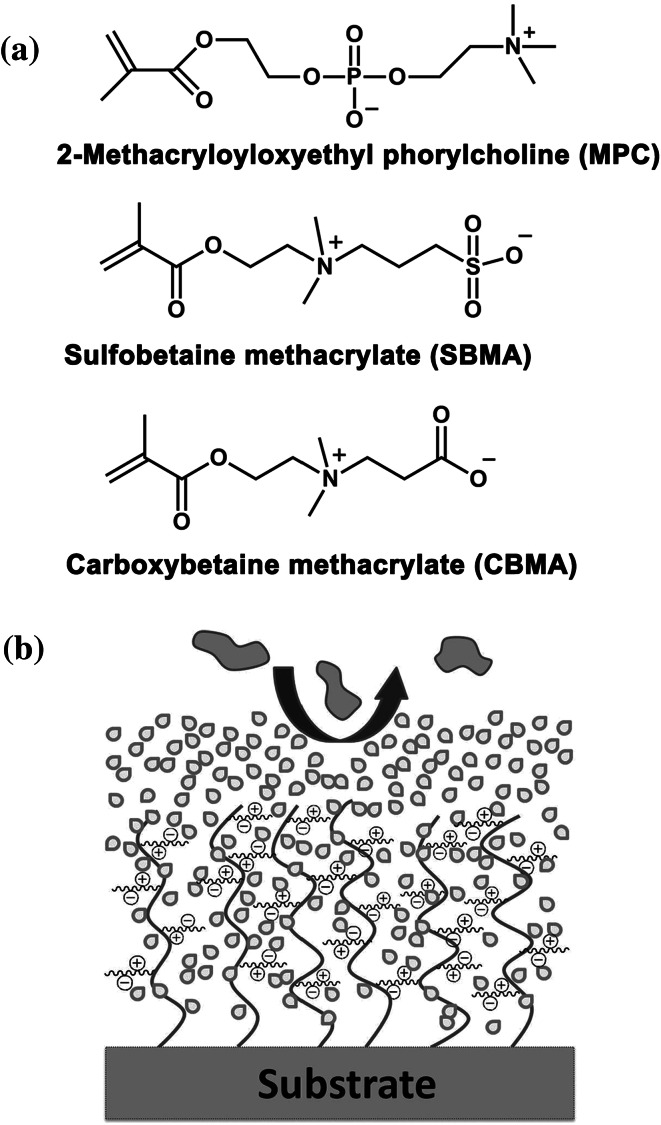
**a** Chemical structures of three types of zwitterionic monomer: 2-methacryloyloxyethyl phorylcholine, sulfobetaine methacrylate, and carboxybetaine methacrylate. **b** Illustration of tight hydration layer mechanism of zwitterionic polymers to surface resistance for nonspecific protein adsorption

In the report of Seo et al. [75], block/random-type amphiphilic copolymers with different compositions of a hydrophilic PC-containing monomer, 2-methacryloyloxyethyl phorylcholine (MPC, Fig. 3a), and hydrophobic dimethylsiloxane (DMS) units were synthesized for a rapid and simple surface modification of PDMS (Table 2). Due to the high affinity of DMS units with the PDMS elastomer, DMS was chosen to work as a stabilizing unit to maximize the hydrophobic interaction of the copolymer with PDMS substrates. For the random-type copolymer, the ratio of DMS units should be at least 70 % to stably immobilize the polymer on the PDMS surface in aqueous media. Suppressed bovine serum albumin (BSA) adsorption was found on a PDMS surface coated with such MPC-containing copolymers. Compared to a bare PDMS surface, L929 fibroblasts showed very low adhesion on the modified surface.

**Table 2:** Polyzwitterion-based coatings for PDMS devices

Coating methods	Chemical structures	Description	Ref.
Physical adsorption		Materials were synthesized as block and random-type amphiphilic copolymers and adsorbed on PDMS by hydrophobic interaction	[75]
		Copolymers of SBMA and AA with various compositions were synthesized and attached onto substrates with polyelectrolyte films via electrostatic interaction	[79]
		A triblock copolymer PCB-PPO-PCB was synthesized and coated on hydrophobic substrates by hydrophobic interaction between PPO parts and substrate face	[85]
Covalent bonding		A uniform pCBMA coating was formed by SI-ATRP of CBMA on PDMS, exhibiting long-term hydrophilic stability and anti-fouling ability	[86]

A PC-containing coating can reduce protein fouling effectively, but the phosphoester groups readily undergo hydrolysis [76]. In addition, the synthesis and preparation of MPC is difficult because of its sensitivity to moisture [77]. Therefore, new materials with better stability and longer nonfouling ability are desirable for biomedical applications. Poly(sulfobetaine) (PSB) and poly(carboxybetaine) (PCB) are two materials belonging to polybetaines, in which both cationic and anionic groups are on the same monomer residue, similar to phosphobtaines [78]. However, compared with MPC monomers, SB methacrylate (SBMA, Fig. 3a) and CB methacrylate (CBMA, Fig. 3a) are more stable [77] and easier to deal with, which makes them more attractive for a wide range of practical applications.

Kuo et al. [79] synthesized zwitterionic copolymers of SBMA and acrylic acid (AA) [poly(SBMA-co-AA)] with various compositions (Table 2). These polymers were then immobilized onto polymeric substrates including PDMS, tissue culture polystyrene (TCPS), and polyurethane (PU) with layer-by-layer polyelectrolyte films via electrostatic interaction. Briefly, three layers of polyelectrolytes, namely poly(ethylenimine) PEI, poly(acrylic acid-g-azidoaniline) (PAA-g-Az) and PEI were sequentially first deposited on subtrates. Then, on the top of the trilayered polyelectrolyte base, one layer of poly(SBMA-co-AA) was coated in the dark. Finally, the stability of the resulting multilayer films was strengthened by the UV-cross-linking of azide groups. The experimental results indicated that the surface coated with poly(SBMA56-co-AA44) formed a uniform coating with a high surface SBMA density which led to high efficacy in inhibiting fibrinogen adsorption, platelet adhesion and plasma coagulation.

Among polybetaines, PCB has the longest history [80]. It has been reported that PCB showed undetectable nonspecific protein adsorption (<0.3 ng·cm^−2^) from single-protein solutions [81] or complex media [82, 83], and can effectively resist protein fouling from lysozyme, fibrinogen, and human chorionic gonadotropin [84]. Moreover, it is worth mentioning that PCB has the functionalizable carboxyl group which facilitates the immobilization of biological molecules with special functions. Therefore, PCB-coated surfaces with suitable decoration can resist nonspecific protein fouling and bind specific biomolecules to be a bioactive surface for various biomedical applications. The unique dual-functional features of CB moieties make PCB more attractive than other similar anti-fouling polymers. In a study of Jiang et al. [85], a zwitterionic triblock copolymer, PCB-PPO-PCB (Table 2), was synthesized and coated on different hydrophobic surfaces including PDMS, silanized silica, and a surface plasmon resonance sensor surface, by self-assembly based on hydrophobic–hydrophobic interactions. The PCB-PPO-PCB modified surface was demonstrated to allow specific biomolecule immobilization as well as to possess effective resistance to nonspecific protein fouling from undiluted human blood plasma. Jiang et al. [86] also introduced CBMA to the PDMS surface via the SI-ATRP technique, creating a highly stable coating that suppresses the surface hydrophobic recovery of PDMS. A long-term nonfouling ability of the modified surface, up to 31 days in dry air and >64 days in aqueous conditions, was observed.

Mixed-charge materials such as polyampholyte polymers [87, 88] with separate moieties of opposite charges are equivalent to zwitterionic materials. An anti-fouling surface can be created using such materials when they are uniformly distributed on the substrate surface with balanced charged groups at the molecular level.

It is believed that the hydration layer (Fig. 3b) is mainly responsible for the repelling property of zwitterionic materials. Compared with conventional hydrophilic materials whose surface hydration is formed via hydrogen bonding, charged zwitterionic materials can bind with water molecules more strongly via electrostatically induced hydration. It was reported that a large free water fraction exists near zwitterionic materials such as MPC [89, 90], and that water molecules could be adsorbed and polarized quickly in the PC region [91]. The resulting thick hydration layer allows proteins to stay in a stable conformation when approaching the substrate surface, avoiding irreversible adsorption [89, 90].

Another explanation for the protein-resistance of zwitterionic materials is that the organized bilayer of plasma lipids formed on the surface acts as a shield to prevent protein adsorption [92, 93]. However, this cannot explain the existence of protein resistance when a modified surface is exposed to biological fluids without plasma lipids. So similar to PEG, mobility and flexibility of the zwitterionic polymer are considered to contribute to protein resistance [94, 95]. Thus, surface modification parameters such as the chain length of polymer, modification density, and temperature should be taken into account when preparing a nonfouling surface with zwitterionic polymers.

### Other Protein-Resistant Coatings

#### Saccharide-Based Coatings

Studies on biomaterials have proven that saccharides can be utilized as potential alternatives to PEG [96]. They have good biocompatibility and low toxicity and can be functionalized for specific biomedical applications. Saccharides have been used to coat material surfaces including PDMS for biomolecule separation, immunoassays, and protein anti-fouling [97–99]. Yang et al. [100] modified a PDMS surface with polysaccharides such as carboxymethyl cellulose, carboxymethyl β-1,3-dextran, and alginic acid by photocatalyzed covalent linking. A protein adsorption assay showed that carboxymethyl β-1,3-dextran- and alginic-acid-coated PDMS surfaces reduced the adsorption of chicken egg albumin and negatively charged BSA, but increased the adsorption of positively charged RNase-A and lysozyme. Carboxymethyl-cellulose-modified PDMS, however, showed protein-repelling ability for both types of protein. The repelling mechanism was not addressed in the report. In another report, n-Dodecyl-b-D-maltoside (DDM), an alkyl polyglucoside, (a type of nonionic surfactant), was used to modify a PDMS surface as its alkyl part can adsorb strongly on a hydrophobic surface [101]. The formed monolayer of DDM caused the PDMS surface to become hydrophilic and nonionic, which effectively minimized nonspecific protein adsorption.

#### Polyhydroxy-Polymer-Based Coatings

Compared with polymers with less hydrogen bond acceptors, hydroxyl-enriched polymers can bond more water molecules through abundant hydrogen bonds, leading to their high hydrophilicity. The hydration layer formed around the materials is strongly correlated with their anti-fouling performance. Wu et al. created multilayer poly(vinyl alcohol) (PVA) coatings on PDMS microfluidic chips [102]. The self-assembled PVA multilayer was further heat-treated at 140 °C for immobilization. They found 88 % hydrolyzed PVA adsorbed more strongly on the oxygen-plasma-pretreated PDMS surface than did 100 % hydrolyzed PVA. The PVA coating prevented both basic and acidic protein adsorption. Other polyhydroxy polymers, such as poly(2-hydroxyethyl methacrylate) [103] and dendritic/hyperbranched polyglycerols [104, 105], also exhibit ultra-low protein fouling properties, which are comparable to or better than those of PEG-based coatings.

#### Amide-Containing-Hydrophilic-Polymer Based Coatings

It has been reported that polymers with amide groups in side chains such as polyacrylamide can reduce protein adsorption and expedite electrophoretic separations of proteins [106–108] due to their high hydrophilicity. Wirth et al. grafted polyacrylamide onto a PDMS surface by ATRP to achieve resistance to the irreversible adsorption of lysozyme [109, 110]. In comparison with untreated PDMS, the polyacrylamide-coated surface exhibited about 20 times lower protein adsorption [109]. The coated surface was stable with respect to hydrophilicity for over 1 month. A polyacrylamide-modified PDMS surface displayed a reduced adsorption of peptides [111] and a temperature-responsive capability [112] because of the reversible hydrogen bonding between amide groups and water molecules that depends on temperature. Hydrophilic poly(N-vinylpyrrolidone) (PVP), a special biocompatible amide-containing polymer, also showed good anti-fouling properties against protein adsorption as well as cell adhesion when grafted on glass [113], silicon [114], and PDMS [115] surfaces. As both the backbone and pyrrolidone side groups of PVP are less flexible than PEG chains, the anti-fouling ability of PVP is considered to be associated with the water barrier formed by polar pyrrolidone units. In addition to the polymers of amide groups in side chains, peptide-like polymers with amide groups in the backbone have been demonstrated as good fouling-resistant materials for surface modification [116–118]. However, the anti-fouling mechanisms are unknown. In a very recent report [119], poly(dimethylaminoethyl methacrylate)-grafted PDMS (PDMS-QPDMAEMA) surfaces were demonstrated to have reduced protein and cell contamination and antimicrobial ability when the tertiary amino group was quaternized by ethyl bromide.

#### Fluoro-Containing Coatings

Fluorinated-polymer-based coatings can modify host surfaces to produce minimized surface energy and low surface tension [120–125], and thus resist fouling. Fouling-release coatings were formed through surface self-organization of films made from blends of a fluorinated/siloxane copolymer with a PDMS matrix [126, 127]. Such films incorporated the low surface tension of fluorinated polymers and the low elastic modulus character of PDMS, showing a decrease in the settlement of barnacles and lower adhesion strength of young algae *Ulva* compared to those of the siloxane control. Horton et al., used commercially available perfluorinated alkane perfluoro-1,1,2,2-tetrahydrooctyl-1-triethoxysilane to modify PDMS by grafting it onto the oxidized surface of a PDMS substrate [128, 129]. The fluorinated PDMS allowed the selective adsorption of fluorous-tagged peptides such as cytochrome c, carbonic anhydrase, insulin, and ubiquitin. It thus has the potential for the detection of fluorous-labeled proteins and peptides.

## Conclusion

The fouling of proteins from biological fluids is a significant challenge for the use of biomedical devices. Preventing or controlling nonspecific protein adsorption can ensure good performance of devices and prolong their service period. Surface coating or surface modification is an effective way of generating new properties on material surfaces without changing the bulk feature of the host material.

Polyhydrophilic and polyzwitterionic materials are two typical types of anti-fouling polymer for surface coatings. Hydrophilic PEG-containing polymers have been demonstrated to have resistance to nonspecific protein adsorption and cell adhesion. However, the long-term stability of PEG-based surface coatings in a biological environment still needs to be improved for practical applications because PEG is subject to oxidation when exposed to most biochemical solutions. Zwitterionic polymers are very promising as next-generation anti-fouling and antimicrobial biomaterials due to their outstanding anti-fouling properties. Steric repulsion and hydration layer mechanisms have been proposed to explain the anti-fouling ability of these materials. However, controlling protein adsorption at a low level is still difficult to achieve when the surface is exposed to undiluted serum or plasma. Protein fouling in real-word applications is more complex not only because protein structures and conformation can vary in different biological environments, but also due to the diversity of proteins (different MWs and components) and the unknown influence among different proteins and/or compositions in biological media [130]. Therefore, the underlying principles of protein fouling and adsorption process under complex conditions should be further studied to allow the development of low-fouling surfaces for biomedical devices.
